# Identification of plasmids in avian-associated *Escherichia coli* using nanopore and illumina sequencing

**DOI:** 10.1186/s12864-023-09784-6

**Published:** 2023-11-21

**Authors:** Haley Sanderson, Madeline C. McCarthy, Chinenye R. Nnajide, Jessica Sparrow, Joseph E. Rubin, Jo-Anne R. Dillon, Aaron P. White

**Affiliations:** 1 Vaccine and Infectious Disease Organization (VIDO), University of Saskatchewan, Saskatoon, SK Canada; 2 Department of Biochemistry, Microbiology and Immunology, University of Saskatchewan, Saskatoon, SK Canada; 3 Current address: Department of Biochemistry and Biomedical Sciences, McMaster University, Hamilton, ON Canada; 4 Department of Veterinary Microbiology, University of Saskatchewan, Saskatoon, SK Canada

**Keywords:** Plasmids, Avian pathogenic escherichia coli, Nanopore sequencing, Illumina sequencing, Hybrid assembly, MOB-suite

## Abstract

**Background:**

Avian pathogenic *Escherichia coli* (APEC) are the causative agents of colibacillosis in chickens, a disease which has significant economic impact on the poultry industry. Large plasmids detected in APEC are known to contribute to strain diversity for pathogenicity and antimicrobial resistance, but there could be other plasmids that are missed in standard analysis. In this study, we determined the impact of sequencing and assembly factors for the detection of plasmids in an *E. coli* whole genome sequencing project.

**Results:**

Hybrid assembly (Illumina and Nanopore) combined with plasmid DNA extractions allowed for detection of the greatest number of plasmids in *E. coli*, as detected by MOB-suite software. In total, 79 plasmids were identified in 19 *E. coli* isolates. Hybrid assemblies were robust and consistent in quality regardless of sequencing kit used or if long reads were filtered or not. In contrast, long read only assemblies were more variable and influenced by sequencing and assembly parameters. Plasmid DNA extractions allowed for the detection of physically smaller plasmids, but when averaged over 19 isolates did not significantly change the overall number of plasmids detected.

**Conclusions:**

Hybrid assembly can be reliably used to detect plasmids in *E. coli*, especially if researchers are focused on large plasmids containing antimicrobial resistance genes and virulence factors. If the goal is comprehensive detection of all plasmids, particularly if smaller sized vectors are desired for biotechnology applications, the addition of plasmid DNA extractions to hybrid assemblies is prudent. Long read sequencing is sufficient to detect many plasmids in *E. coli*, however, it is more prone to errors when expanded to analyze a large number of isolates.

**Supplementary Information:**

The online version contains supplementary material available at 10.1186/s12864-023-09784-6.

## Background

*Escherichia coli* is a Gram-negative bacillus that is a common inhabitant in the intestines of warm-blooded animals, and can cause a wide range of diseases in poultry that fall under the term colibacillosis [1]. This includes localized infections of the reproductive tract, yolk sac, and umbilical stump in chicks, as well as septicemia [1, 2]. Avian pathogenic *E. coli* (APEC) are thought to be poultry commensal strains that escape the intestinal niche to cause disease in other parts of the body [3]. However, APEC are difficult to characterize due to the great diversity of strains recovered from diseased broilers [4]. The genetic plasticity and promiscuity of *E. coli* allows them to play an important role in the spread of antimicrobial resistant genes [5]. Multi-drug resistance is common among *E. coli* from avian sources, irrespective of host disease status [6]. Many large plasmids contribute to the diversity of APEC as a whole, allowing for genes related to virulence and antimicrobial resistance to spread and allowing the organism to better adapt to environmental conditions [7]. APEC plasmids are thought to play a role in the transition from commensal to pathogenic isolate [7]. For instance, the curing or introduction of a plasmid can impact the lethality of APEC infections [7, 8]. In addition to APEC, plasmids have been shown to be important for other *E. coli* pathotypes [9].

Plasmids are an important aspect of the genome for the epidemiological study of pathogens and antimicrobial resistance. Plasmids typically include a “backbone” of core genes that are associated with plasmid-specific functions, such as replication and mobility, and are generally conserved among broadly related plasmids of the same family [10]. Plasmids also carry accessory genes, which often confer clinical- or environmental-relevant traits such as virulence, heavy metal and antimicrobial resistance [11]. These accessory genes are frequently spread due to their presence on transmissible plasmids, which can enable bacteria to evolve rapidly under environmental pressure [12, 13]. An example of this is the spread of genes encoding carbapenem and colistin resistance [14].

The most common technology used for pathogen and AMR surveillance is Illumina sequencing by synthesis, which can generate millions of low-error (0.1%) short (100-300 bp) paired-end reads [15]. However, it is difficult to reconstruct plasmids using only Illumina short reads, due to the presence of repetitive regions that can exceed the length of the short reads. The use of longer reads (8-10 kb or greater) add value when reconstructing plasmids and other mobile genetic elements. However, the higher error rate (5–15%) of Oxford Nanopore Technology (ONT) sequencing and high startup costs for PacBio Single Molecule sequencers remains a challenge in using long read sequencing for characterizing genomes, though these barriers continue to reduce over time [15, 16]. Hybrid assembly, combining short-read and long-read sequencing approaches, has been shown to be effective in reconstructing accurate, contiguous genomes, including plasmids. Long reads can provide the plasmid structure and span repetitive regions, while the short reads can be used to correct any sequencing errors [17].

With the increased accessibility and decreasing cost of sequencing technologies, the problem is no longer the availability of sequencing data but the ability to process and analyze that data [18]. There are many bioinformatic tools designed to assemble genomes that are sequenced using short and long read technologies. Many studies have tried to determine which technologies are best for both generating complete assemblies and for the detection of plasmids. Unicycler, a genome assembler, has been found to produce good quality assemblies from short read sequencing and has been found to recover plasmids also [19]. Nanopore and Illumina reads have been found to generate the best hybrid assemblies when using Unicycler without filtering the reads for quality and length [15]. In terms of producing complete genomes from long read sequencing, Flye, a long read genome assembler, has been found to be the superior assembler due to reduced run times and producing high-quality assemblies [20].

In this study, we sought to determine the most efficient and effective way to characterize the plasmidome of 19 avian-associated *E. coli* strains. We compared three methods for sequencing the plasmids, using six plasmid extraction kits, long read whole genome sequencing and combining long and short read (i.e., hybrid) DNA sequencing. The principles of what we found can be applied to any *E. coli* group or pathotype. The identification of plasmids within a genome assembly is the first step to characterize the potentially important role of these plasmids in AMR, virulence and niche adaptation for APEC and other *E. coli* pathotypes.

## Results

### How well do plasmid extraction kits work for detecting *E. coli *plasmids?

Two avian *E. coli* isolates were chosen to test the recovery of plasmids based on six commercial plasmid extraction kits (Table 1). Purified DNA samples from *E. coli* 4957-3S1 and 4957-C3 were prepared for long-read sequencing using the Nanopore Ligation Sequencing Kit, hereafter referred to as “Ligation”. In the Ligation protocol, adapters are ligated to nicked or linearized DNA fragments. We hypothesized that during routine plasmid purification some proportion of the circular plasmid DNA molecules would become ‘nicked’ and could have barcode primers ligated directly without performing additional fragmentation steps. In practice, fresh plasmid samples had fewer nicked DNA molecules than older samples (i.e., stored frozen for > 1 week), and library preparation resulted in a relatively low number (i.e., < 10,000) of Nanopore raw reads per sample. Going forward, to ensure that a uniform number of reads was generated, we incorporated a sonication step to intentionally nick or linearize the circular DNA fragments. We also sequenced plasmid extraction samples using the Nanopore Rapid Barcoding Kit, hereafter referred to as “Rapid”, which incorporates a transposon-based enzymatic fragmentation step, removing the need for sonication.

**Table 1:** Detection of plasmids in two avian-associated *E. coli* isolates using commercial plasmid extraction kits

		*E. coli* isolate 4957-3S1				*E. coli* isolate 4957-C3						
Plasmid Extraction Kit^a^	Sequencing Kit^b^	AB241^c^	AB690	AA175	AC044	AB690	AB241	AA176	AA378	AB526	AE638	AG799
A	Ligation	+	+	-	-	+	-	-	-	-	-	-
A	Rapid	+	+	-	-	+	+	-	-	-	-	-
B	Ligation	+	+	-	-	+	+	-	-	-	-	-
B	Rapid	+	+	-	-	+	-	-	-	-	-	-
C	Ligation	+	+	-	-	+	-	-	-	-	-	-
C	Rapid	+	+	-	-	+	-	-	-	-	-	-
D	Ligation	+	-	-	-	+	-	-	-	-	+	-
D	Rapid	+	-	-	-	+	-	-	-	-	-	-
E	Ligation	-	-	-	-	+	-	-	-	-	-	-
E	Rapid	+	-	-	-	+	-	-	-	-	-	-
F	Ligation	-	-	-	-	-	-	-	-	-	-	+
F	Rapid	+	-	-	-	+	-	-	-	-	-	-
Combinations^d^												
A/B	Ligation	+	+	-	-	+	-	-	-	-	-	-
A/B	Rapid	+	+	-	-	+	+	-	-	-	-	-
A/C	Ligation	+	-	-	-	+	-	-	-	-	-	-
A/C	Rapid	+	+	-	-	+	-	-	-	-	-	-
A/D	Ligation	+	+	-	-	+	-	-	-	-	-	-
A/D	Rapid	+	-	-	-	+	-	-	-	-	-	-
A/E	Ligation	+	+	-	-	+	-	-	-	-	-	-
A/E	Rapid	+	-	+	-	+	+	-	-	-	-	-
A/F	Ligation	+	-	-	-	+	-	-	-	-	-	-
A/F	Rapid	+	+	-	-	+	+	-	-	-	-	-
B/C	Ligation	+	+	-	-	+	-	-	-	-	-	-
B/C	Rapid	+	+	-	-	+	-	-	-	-	-	-
B/D	Ligation	+	+	-	-	+	-	-	-	-	-	-
B/D	Rapid	+	-	-	-	+	-	-	-	-	-	-
B/E	Ligation	+	+	-	-	+	-	-	-	-	-	-
B/E	Rapid	+	-	-	-	+	-	-	-	-	-	-
B/F	Ligation	-	-	-	-	+	-	-	+	-	-	-
B/F	Rapid	+	+	-	-	+	-	-	-	-	-	-
C/D	Ligation	-	+	-	-	+	-	-	-	-	-	-
C/D	Rapid	+	-	-	-	+	-	-	-	-	-	-
C/E	Ligation	+	-	-	-	+	-	-	-	-	-	-
C/E	Rapid	+	-	-	-	+	-	-	-	-	-	-
C/F	Ligation	+	-	-	+	+	-	-	-	-	+	-
C/F	Rapid	+	-	-	-	+	-	-	-	-	-	-
D/E	Ligation	+	+	-	-	+	-	+	-	-	+	-
D/E	Rapid	+	-	-	-	+	-	-	-	-	-	-
D/F	Ligation	-	-	-	-	-	-	-	-	-	-	-
D/F	Rapid	+	-	-	-	-	-	-	-	+	-	-
E/F	Ligation	+	-	-	-	-	-	+	+	-	-	-
E/F	Rapid	+	-	-	-	+	-	-	-	-	-	-

^a^Plasmid-containing samples were prepared from the two *E. coli* isolates using commercial plasmid extraction kits: A – GenElute plasmid miniprep kit (#PLN70; Millipore Sigma); B – NucleoSpin plasmid mini kit (#740588.50; Machery-Nagel); C – Presto mini plasmid kit (#PD100; Geneaid); D – Monarch plasmid miniprep kit (#T1010S; New England Biolabs); E – Plasmid midi kit (#12,143; Qiagen); and F – GeneJET plasmid miniprep kit (#K0502; ThermoFisher)

^b^Sequencing libraries from each plasmid sample were generated using the Nanopore Ligation or Rapid kits with sequencing performed on the Nanopore MinION Mk1C.

^c^Plasmid clusters were detected by MOB-suite software (v3.1.0) in each finished DNA assembly from each *E. coli* isolate. The metadata corresponding to each ID listed is in Table S2

The results from each plasmid extraction kit were analyzed individually, and in all pairwise combinations, representing a total of 37 assemblies for each sequencing kit and *E. coli* strain. For 4957-3S1, plasmid cluster AB241 was identified in 33 of 37 assemblies and AB690 was identified in 19 of 37 assemblies (Table 1). For 4957-C3, plasmid cluster AB690 was identified in 36 of 37 assemblies (Table 1). Combining the assemblies from the Ligation and Rapid kits led to the detection of 2 additional plasmid clusters for 4957-3S1 and 6 additional plasmid clusters for 4957-C3 (Table 1). In general, detection of these additional plasmids was not consistent, and neither the Ligation nor Rapid kit were clearly superior to each other. The sequencing output and parameters from the Ligation and Rapid kits were also similar (Table S1). We chose to use the Ligation kit with sonication to sequence any additional plasmid extraction samples.

### Detection of *E. coli* Plasmids as part of a whole genome sequencing project

We performed a standard WGS workflow on 19 *E. coli* isolates, including 4957-3S1 and 4957-C3, with plasmid identification using the MOB-suite program [21]. We tested the influence of three technical parameters: 1) assembly type: hybrid (Illumina + Nanopore) or long read (Nanopore); 2) sequencing kit: Ligation or Rapid sequencing kits; and 3) filtering: if short DNA reads from Nanopore were filtered out or not prior to assembly. We also performed Nanopore sequencing of plasmid DNA samples, purified from each isolate using two commercial plasmid extraction kits.

**Fig. 1:**
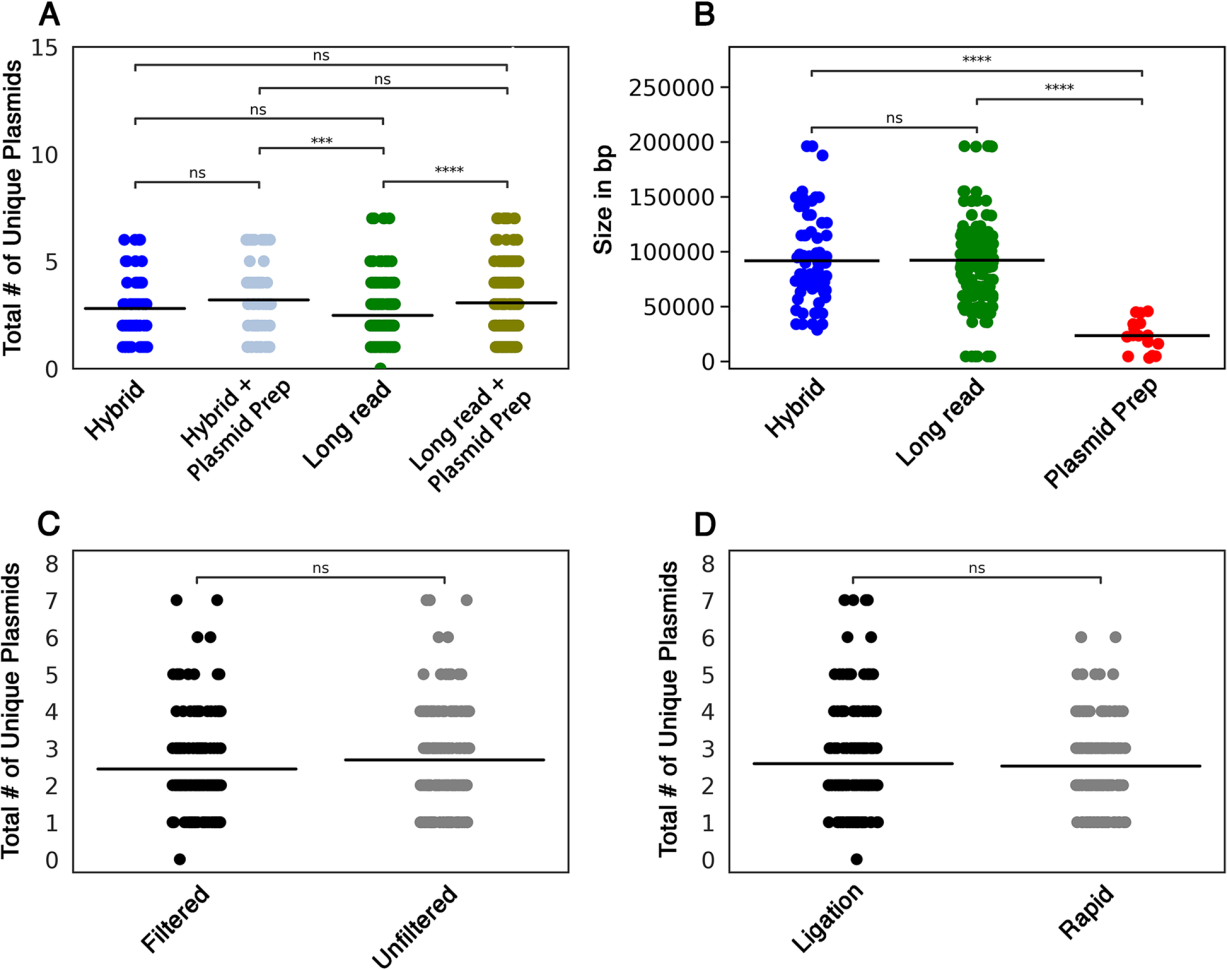
Hybrid (Illumina + Nanopore) and long read (Nanopore) sequencing to detect* E. coli *plasmids.  DNA sequence assemblies were prepared from 19 different E. coli isolates and screened for plasmids using MOB-suite software. Strip plots are shown for the total number of unique plasmids detected in: (**A**) Hybrid and long read assemblies (filtered and unfiltered; Nanopore Ligation and Rapid kits) with or without plasmid prep assemblies; (**C**) Hybrid and long read assemblies, generated using Nanopore Ligation and Rapid kits, where raw reads were filtered (i.e., > 1000 bp cut-off) or unfiltered prior to assembly; and (**D**) Hybrid and long read assemblies (filtered + unfiltered) generated using Nanopore Ligation or Rapid kits. (**B**) The sizes of plasmids detected in all hybrid, long read and plasmid prep assemblies. Bars in (A, B, C, D) represent the mean values. The significance of relationships was determined using t-tests ( *P*  > 0.05, ns; *P*  < 0.05, *; *P*  < 0.001, ***; *P*  < 0.0001, ****)

On a strain-by-strain basis, more plasmids were detected in the hybrid assemblies than the long read assemblies, although the difference in mean plasmid number between 19 *E. coli* strains was not statistically significant (Fig. 1A). When sequence assemblies from the plasmid extraction kits were included, the mean number of detected plasmids was increased for both hybrid and long read assemblies, although the increase was only significant when added to the long read assemblies (Fig. 1A). This result indicated that the plasmids identified from the extraction kits were unique to those detected by WGS assembly. Consistent with this, the plasmids identified by plasmid extraction were significantly smaller in size than the plasmids identified by hybrid or long read assemblies (Fig. 1B). The DNA fragments corresponding to these smaller plasmids were not present in the WGS procedure, since performing assemblies with unfiltered reads (i.e., including all small fragments) did not significantly change the number of detected plasmids (Fig. 1C). The mean number of identified plasmids also did not vary significantly between use of the Ligation or Rapid kits (Fig. 1D).

We compared hybrid and long read assemblies using Quast [22] to determine if the assemblies differed in quality. All four hybrid assemblies (HLF, hybrid ligation filtered; HLUF, hybrid ligation unfiltered; HRF, hybrid rapid filtered; HRUF, hybrid rapid unfiltered) were successful with no significant differences in quality parameters including N50 (Figure S1A), number of contigs (Figure S1B), largest contig (Figure S1C), total length of assembly (Figure S1D) and GC content (Figure S1E). The quality of the long read assemblies was more variable and influenced by the type of sequencing kit used, with significant differences in the N50 (Figure S2A), the number of contigs (Figure S2B), the size of the largest contig (Figure S2C), and the GC content (Figure S2E). Filtering (i.e., removal of < 1000 bp fragments) prior to assembly impacted the number of contigs (Figure S2B) and the GC content (Figure S2E). The last parameter tested for long read assembly was the polishing step. There was no significant difference in the number of plasmids identified if the assemblies were left unpolished, polished with long reads or polished with long-reads and short reads, a form of hybrid assembly where the long reads were assembled first (Figure S3). Our overall conclusion was that hybrid assemblies were less prone to variation based on sequencing and assembly factors.

### Hybrid genome assembly will identify the majority of plasmids in *E. coli*

**Fig. 2:**
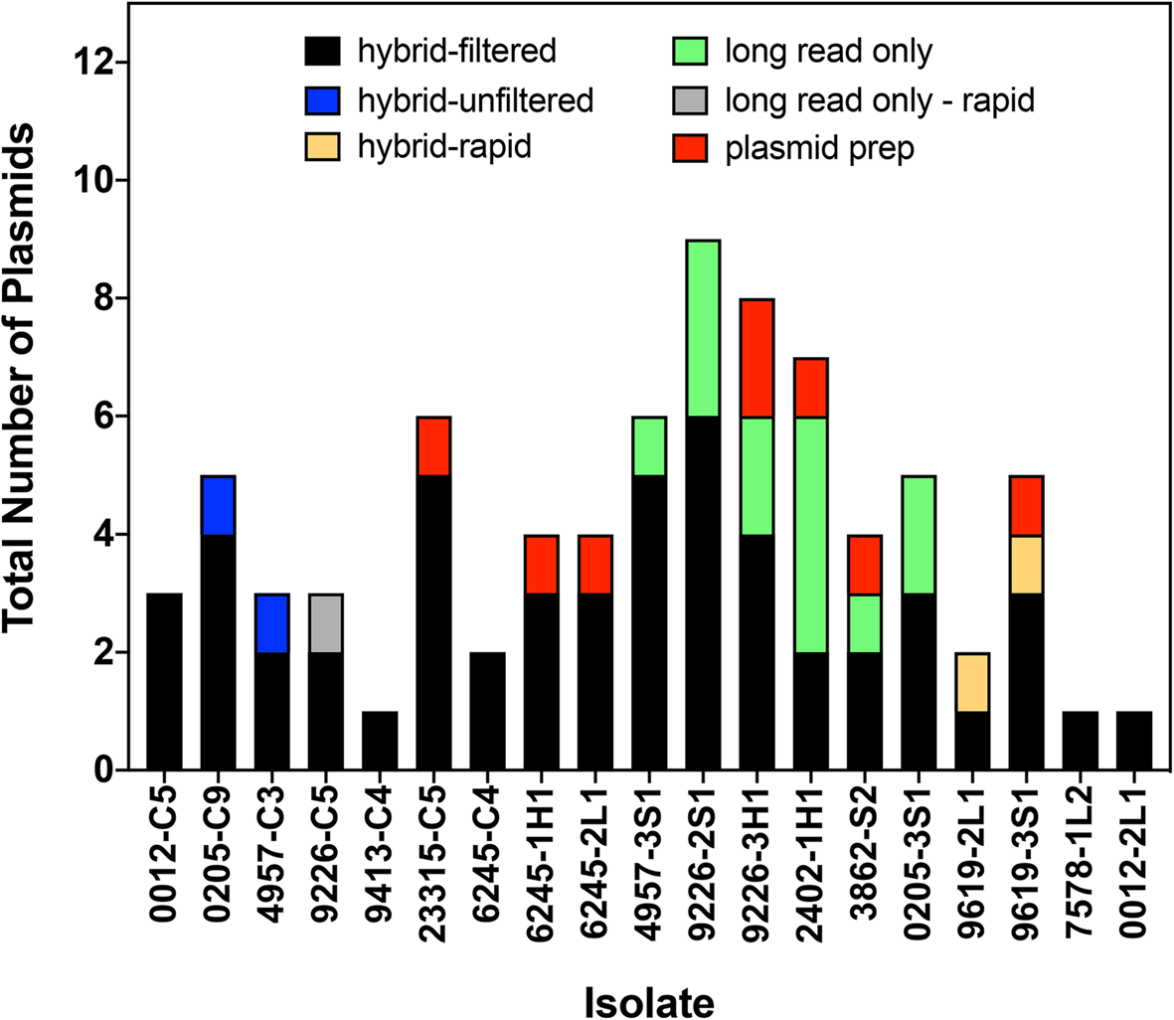
Total numbers of plasmids detected in 19 avian-associated* E. coli. *Bars represent the number of unique plasmid clusters detected in each isolate by MOB-suite software, based on six different combinations of DNA sequencing and assembly: 1) hybrid-filtered, Illumina + Nanopore Ligation with filtering (black); 2) hybrid-unfiltered, Illumina + Nanopore Ligation without filtering (blue); 3) hybrid-rapid, Illumina + Nanopore Rapid with or without filtering (yellow); 4) long read only, Nanopore Ligation with or without filtering (green); 5) long read only – rapid, Nanopore Rapid with or without filtering (grey); or 6) plasmid prep, Nanopore Ligation with or without filtering (red)

The data from all DNA sequencing and assembly approaches were combined, and a total of 79 individual plasmids were detected in 19 *E. coli* isolates. The numbers ranged from 9 plasmids in isolate 9226-2S1 to a single plasmid in isolates 9314-C4, 7578-1L2 and 0012-1L2 (Fig. 2). Fifty-three of the 79 plasmids (i.e., 67%) were detected using the standard WGS hybrid-filtered assembly (Fig. 2; black bars). In contrast, hybrid-unfiltered (blue bars) and hybrid-rapid (yellow bars) assemblies had relatively minor contributions to the total (Fig. 2). Long-read only assemblies detected 14 unique plasmids overall from 7 different isolates (Fig. 2). Sequencing of purified plasmid samples (i.e., plasmid prep) identified a total of 8 plasmids, detected in 7 isolates (Fig. 2). This analysis indicated that a variety of sequencing and assembly approaches is helpful for the detection of all plasmids that may be present in an *E. coli* isolate.

### Distribution of MOB-suite plasmid clusters within *E. coli *isolates

Each plasmid cluster identified by MOB-suite represents a group of similar plasmids that contain key sequence features and can be analyzed together, as determined based on mash distance from reference plasmid sequences [21]. In total, within the group of 19 *E. coli* isolates, 34 different plasmid clusters were identified (see Table S2 for detailed information). Eighteen plasmid clusters were detected in two or more isolates, representing 63 of the 79 total plasmids identified, whereas sixteen plasmid clusters were detected only once (Table 2). Ten of the 18 ‘common’ plasmid clusters were larger, ranging in size from 40,000 to 230,000 bp and containing > 250 ORFs, whereas the eight remaining ‘common’ plasmid clusters were smaller than 20,000 bp and had fewer than 200 ORFs (Table S2). The most common plasmid cluster was AA176 with an average size of 155,753 bp and an average GC content of 50.2%. Plasmids in this cluster had up to 4 incompatibility groups, with IncF1B being the most common, and were classified as conjugative, because of the presence of a mate-pair formation marker and a relaxase (Table S2). In general, each plasmid cluster was genetically similar to plasmids that had been identified before in *E. coli* and closely related species such as *Salmonella enterica*, *Klebsiella pneumoniae*, and *Shigella sonnei* (Table 2).

**Table 2:** Characteristics of different plasmid clusters identified in avian-associated E. coli by MOB-suite software

Plasmid Cluster ID^a^	% G + C	*E. coli* Pathotype / Potential Source^b^	E. coli Isolates^c^	Nearest Neighbour^d^	Average Size (bp)	# ORFs^e^
AA176	50.2	APEC, UPEC	4957-C3, 6245-C4, 4957-3S1, 9226-2S1, 9226-3H1, 2402-1H1, 3862-S2, 0205-3S1	CP033091, CP012113, CP001122, CP031107, KU578032, CP012636, CU928146, CP018994	155,753	941–2165 (1157)
AA474	49.7	UPEC, APEC, *Salmonella enterica*	9413-C4, 23,315-C5, 6245-1H1, 6245-2L1, 9226-2S1, 9226-3H1, 9619-3S1	HE654725, NC_019131, CP024093, KT779550, CP039714, CP032264, CP044183, CP016522, CP019996	104,545	513–2359 (893)
AA175	49.2	APEC	6245-1H1, 6245-2L1, 4957-3S1, 9226-2S1, 9226-3H1, 0012-2L1	CP022732, LC484363	123,558	260–1505 (1240)
AC509	48.5	UPEC, *Salmonella enterica*	23,315-C5, 4957-3S1, 9226-2S1, 2402-1H1, 0205-3S1	CP010175, CP019025, CP032941, CP016519, LT985237	7,917	41–105 (55)
AA378	47.9	UPEC	0205-C9, 4957-C3, 2402-1H1, 0205-3S1	CP012494, CP020522, MH844525, CP022733	95,605	698–827 (751)
AB241	55.6	*Salmonella enterica*	6245-1H1, 6245-2L1, 4957-3S1, 4957-C3	CP038600	2,189	13–46 (17)
AB685	51.7	UPEC, *Klebsiella pneumoniae*	23,315-C5, 9226-2S1, 9226-3H1, 9619-3S1	CP023858, CP026493, CP006785, CP024465	1,914	13–35 (20)
AA281	52.6	APEC	4957-3S1, 9226-2S1, 9226-3H1	CP033633, KR905386, CP018772, KR905389, KR905384	136,115	317–1507 (1245)
AA329	52.1	EHEC	0012-C5, 0205-C9, 9226-C5	NC_013362, CP024284	79,383	398–878 (720)
AB233	41.5	*Salmonella enterica*	23,315-C5, 2402-1H1, 0205-3S1	CP024290, EU219533	40,792	185–516 (253)
AB690	42.3	APEC	4957-C3, 2402-1H1, 0205-3S1	CP005932	6,259	15–92 (53)
AA162	50.4	APEC, *Salmonella enterica*	9226-2S1, 9226-3H1	CP039570, CP011433, CP010318, CP030004, CP012684	7,126	36–115 (52)
AA179	49.4	Unknown Pathotype	9619-2L1, 7578-1L2	CP021198, CP019018	155,108	878–1677 (900)
AA313	50.7	Unknown Pathotype	2402-1H1, 3862-S2	CP024287	53,144	388–529 (444)
AA738	45.6	*Salmonella enterica*	4957-3S1, 9619-3S1	MK169211, CP045449	251,326	1660–2080 (1877)
AB443	46.8	UPEC	0012-C5, 0205-C9	CP035351	7,633	35–132 (44)
AB714	49.0	*Salmonella enterica*	2402-1H1, 3862-S2	CP022453	21,326	157–205 (181)
AC748	47.2	NMEC	0012-C5, 0205-C9	CP030115, CP034961	2,235	10–22 (15)
AA178	49.6	APEC	23,315-C5	CP043952	189,407	1481–1601 (1493)
AA315	48.2	UPEC	9226-C5	KU254579	89,815	711–789 (731)
AA372	42.2	*Salmonella enterica*	9226-2S1	CP028155	60,009	247–508 (450)
AA374	47.4	Unknown Pathotype	6245-C4	CP042894, CP029181	152,238	1125–1228 (1152)
AA476	57.0	STEC	9619-3S1	CP031897	11,442	113 (113)
AA551	51.8	Unknown Pathotype	9226-C5	HE613857	46,517	307–402 (327)
AA664	53.7	*Arthrobacter*	3862-S2	CP011006	4,354	42–43 (42)
AA736	54.6	Unknown Pathotype	9226-3H1	CP018771	17,590	160 (160)
AB229	42.5	Unknown Pathotype	23,315-C5	CP023379	38,502	271–288 (280)
AB238	57.3	*Salmonella enterica*	9619-2L1	AY178821	3,389	12–32 (21)
AC026	52.2	Unknown Pathotype	6245-2L1	CP040923	22,046	221–228 (224)
AC028	46.3	UPEC	9226-2S1	KU043116	14,509	134–137 (136)
AC662	49.1	*Shigella sonnei*	6245-1H1	CP019691	43,325	429 (429)
AD482	48.8	Unknown Pathotype	0205-C9	CP033763	3,343	21–26 (24)
AD669	53.7	ETEC	9619-3S1	CP002733	45,498	363–423 (400)
AF267	47.3	*Salmonella enterica*	9226-3H1	CP022065	21,883	190 (190)

^a^The plasmid cluster ID names were generated by MOB-suite software; each represents a group of related plasmids

^b^The *E. coli* pathotypes: APEC, avian-pathogenic; UPEC, urinary-pathogenic; EHEC, enterohemorrhagic; NMEC, neonatal meningitis-causing; STEC, shiga toxin-producing; ETEC, enterotoxigenic, and Unknown, and other bacterial species listed were associated with “Nearest Neighbour” plasmids and could represent a potential source of the plasmid

^c^Names of the *E. coli* strains in this study where this plasmid cluster ID was detected

^d^Plasmids that were genetically similar to the plasmid cluster IDs are listed by accession numbers to the NCBI nucleotide database

^e^The range in the number of open reading frames (ORFs) in each plasmid cluster (taking into account 8-12 different DNA sequence assemblies for each isolate) is shown with the average number of ORFs in parentheses

### Hierarchical clustering reveals potential biological connection between *E. coli *isolates and plasmid clusters

**Fig. 3:**
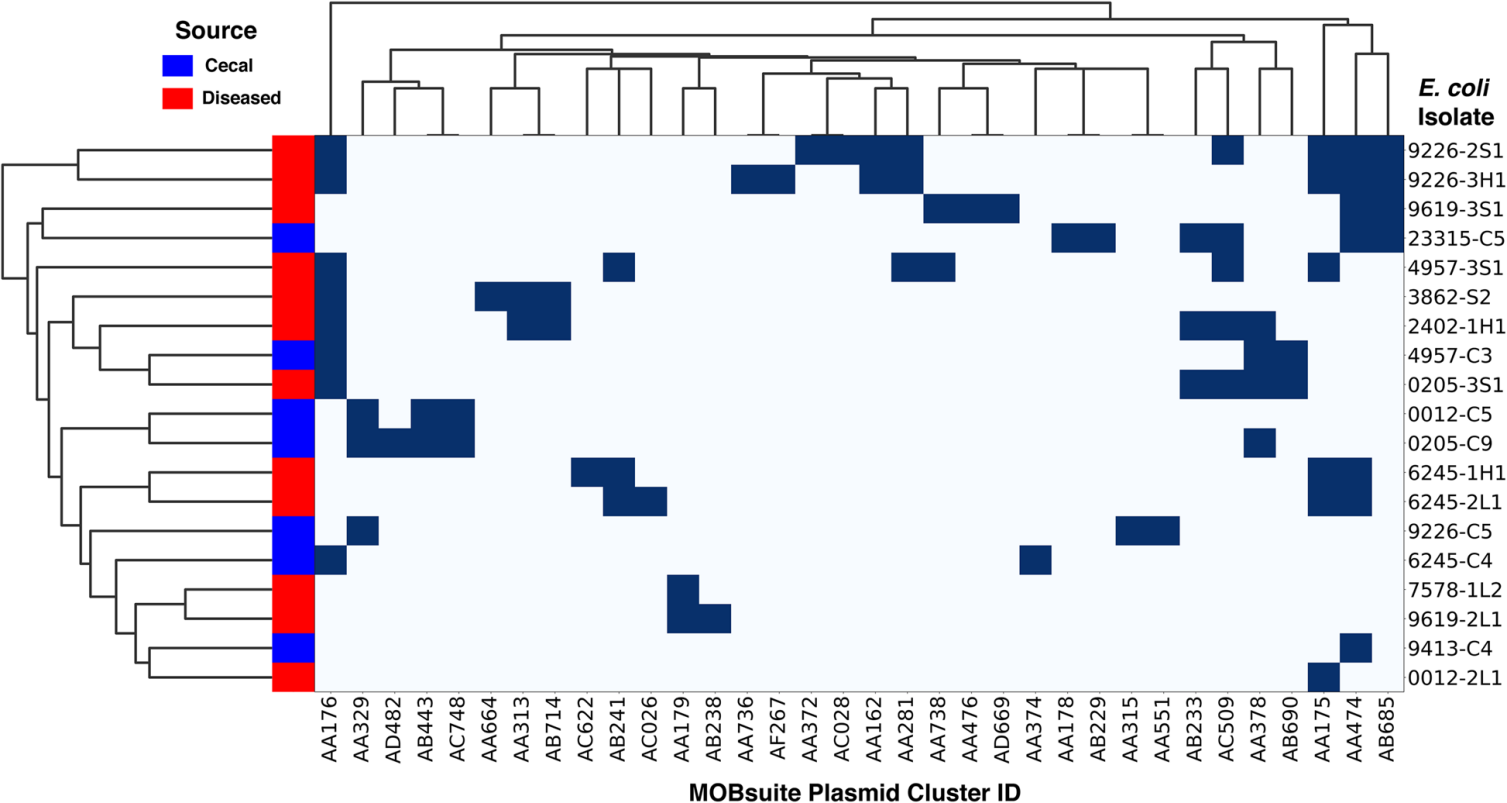
Hierarchical clustering and association of different plasmid clusters with 19 avian-associated *E. coli* strains.  Hierarchical clustering was used to determine the relationship between E. coli isolates (tree shown on left) and plasmid clusters (tree shown on top). Plasmid clusters were identified by MOB-suite software and the names are listed at the bottom. The origin of the E. coli is color coded and shown on the left, and the names of the isolates are shown on the right. Within each row or column, dark blue depicts the presence of the plasmid cluster, whereas light blue depicts its absence

Hierarchical clustering was used to elucidate possible relationships between plasmids, *E. coli* strains and colibacillosis outbreaks. The 19 strains analyzed here consisted of 12 “disease” isolates obtained from internal organs of broilers that died of colibacillosis and 7 “healthy” isolates obtained from the cecal contents of healthy broilers from the same flocks. There was not a lot of overlap in plasmid content between disease-causing and cecal isolates. Disease-causing *E. coli* isolates 9226-2S1 and 9226-3H1, which came from different birds in the same outbreak, clustered together and shared six plasmid clusters (Fig. 3). Cecal isolate 9226-C5, which came from another bird within the same flock, had no plasmids in common with these two disease isolates. The same trend was observed for disease-causing isolates 6245-1H1 and 6245-2L1, which had three plasmid clusters in common, but none that were shared with isolate 6245-C4 (Fig. 3). In three other instances where disease and cecal isolates from the same flocks were analyzed, only one out of 8 plasmid clusters were shared between 4957-3S1 and 4957-C3, one out of 9 plasmid clusters were shared between 0205-3S1 and 0205-C5, and no clusters were shared between 0012-2L1 and 0012-C5 (Fig. 3). In contrast, cecal isolates 0012-C5 and 0205-C9 shared three out of 5 plasmid clusters. There were also plasmid pairs that clustered together across multiple farms, such as AA474 and AB685, AB313 and AB714, and AB443 and AC748 (Fig. 3; Table 2). We concluded from this analysis that there are likely to be biological relationships between strains, strain sources and plasmid clusters that will be more apparent when compared over a larger group of strains [23].

## Discussion

Hybrid genome assemblies, based on a combination of long-read and short-read DNA sequencing, were the most efficient at detecting plasmids carried by *E. coli*. Hybrid assemblies were consistent in quality despite changes to sequencing methods or assembly parameters. Long read only assemblies produced similar results, but the assemblies had more variation based on changes to the sequencing kits used or whether the raw reads were filtered or not prior to assembly. Therefore, the use of long read assemblies would not be as consistent as hybrid assemblies for the detection of plasmids, when analyzing a larger number of isolates. These same principles can likely be applied to any *E. coli* genome sequencing project, including strains from different habitats or representing different pathotypes [24]. For APEC, many large plasmids have been associated with virulence and antimicrobial resistance [25–28], and these are the group of plasmids that were routinely detected within the WGS workflow. We concluded that hybrid WGS is the best option for screening large numbers of genomes and defining the plasmidome of *E. coli*.

Commercial plasmid DNA extraction kits were generally poor in capturing the *E. coli* plasmidome. All six extraction kits that we tested purified smaller sized plasmids, but the different kits did not consistently detect the same group of plasmids. This result was unexpected, and we took the approach to combine two different plasmid extraction kits to perform any further sequencing. Many of the small plasmids detected in the plasmid extraction samples were missed by routine whole genome sequencing. The most likely explanation for this difference in plasmid recovery was due to the size selection step during genomic DNA purification as well as part of the processing and sequencing steps for Nanopore sequencing [29]. The addition of plasmid DNA extractions to the hybrid assemblies did increase the number of plasmids detected within individual strains but did not significantly increase the average number of plasmids detected across the group of 19 strains. However, it did allow for detection of a broader size range of plasmids in each isolate. It should be noted that small plasmids detected by plasmid DNA extractions (i.e., < 20kbp), can be useful for cloning and other biotechnology applications [30–32]. We concluded that plasmid DNA extractions represent a complementary approach to WGS but may not be necessary depending on the goals of the *E. coli* genome sequencing project.

Combining all sequencing approaches (i.e., hybrid, long read, plasmid prep) and assembly parameters (i.e., filtering, polishing) yielded an overall total of 79 plasmids from the group of 19 *E. coli* isolates analyzed. Greater than 60% of plasmids were identified by hybrid WGS only, but this technique did not capture many plasmids under 30 kbp in size. Wick et al. [33] previously reported that the Nanopore Rapid kit was more favorable when trying to detect smaller plasmids, but we did not observe this relationship in our study. When looking at the plasmid clusters detected by each assembly method, differences between the methods became more apparent. Different assembly parameters (i.e., filtering, sequencing kit, assemblers) can allow MOB-Suite to detect specific plasmid clusters in some assemblies and not in others. The program Flye, which is used for long read only assemblies, and Canu, which we used for plasmid DNA extraction assemblies, can sometimes create multiple concatemers of a contig that can influence the assignment of plasmid clusters by MOB-Suite. In our study, filtering the long reads did not impact the overall quality of hybrid assemblies; however, it can impact the assignment of plasmid clusters by MOB-Suite. When combining the results from multiple sequencing and assembly approaches, additional plasmid clusters may represent true plasmids but could also be the result of subtle differences in assembly. Bioinformatic detection of plasmids, using MOB-suite or similar programs, is always a prediction that needs to be verified through laboratory experiments. However, we can conclude from our analysis that filtering to remove short and poor quality long reads for hybrid assemblies yields the best representation of the genome and its plasmid content.

One goal in trying to comprehensively identify *E. coli* plasmids is to determine if biological connections exist between specific plasmids and different sources of *E. coli* isolates. Hierarchical clustering performed on the small subset of 19 strains analyzed here revealed that disease-associated strains from different birds from the same colibacillosis outbreak shared a majority of plasmid clusters. For example, plasmid clusters AA175, AA474, AB685, AA162, and AA281 were detected in *E. coli* 9226-2S1 and 9226-3H1. This could simply reflect a close genetic relationship between the two disease strains, but it could also reflect disease-causing *E. coli* within the same broiler farm sharing the same plasmidome, an idea first proposed by Olsen et al. [27]. In addition, there were also several plasmid clusters that were detected together in cecal and disease-causing isolates from different broiler farms, perhaps due to a genetic link between the individual plasmids. We consider any of these potential plasmid or *E. coli* strain connections as preliminary because we have not yet catalogued the gene content on the plasmids. Any trends identified here should also become more obvious when the analysis is applied across a larger group of isolates. Although it is well established that plasmid DNA contributes substantially to the diversity of avian-associated *E. coli* [24, 27, 34], we detected 34 unique plasmid clusters and an average of 4 plasmids per isolate, which was a higher number than we expected.

Plasmid carriage has been related to the emergence of different *E. coli* pathotypes [34] and could potentially explain how pathogenic strains (such as APEC) can emerge from divergent genetic backgrounds [3]. Furthermore, whole genome sequencing is becoming the standard technique for strain tracking [35] and AMR surveillance [36]. From our study, it is clear that comprehensive plasmid identification requires a significant investment of time, energy and money. For instance, it may be necessary to include plasmid DNA extraction with hybrid WGS assemblies for the analysis of the plasmidome if the goal of the analysis is to analyze all plasmids (or as many as possible) in an isolate. Our results also emphasized the importance of generating multiple assemblies to get closer to comprehensive recovery of plasmids. Similar to other bacterial species (i.e., *Enterococcus faecium* [37]), the best characterized examples of plasmids driving adaption and evolution are with AMR, such as APEC strains in China harboring plasmids that carry genes for resistance to colistin and other antibiotics increasing the likelihood that these strains will survive antimicrobial treatments [38, 39]. Plasmids have also been shown to contain important virulence genes [24, 26] as well as genes with roles in biofilm formation and the interaction of *E. coli* with intestinal epithelial cells [27]. More work with mobile genetic elements is needed to understand their role *E. coli* biology and evolution.

## Conclusions

We determined that comprehensive plasmid identification in *E. coli* required hybrid genome assembly combined with plasmid extraction kits. In some cases, long read only assemblies may provide similar results, but it is more dependent on methodological choices for its coverage of plasmids and overall genome quality. The inclusion of plasmid DNA extractions in addition to hybrid assemblies is required only if small plasmids (less than 50kbp) are of interest. For avian-associated *E. coli*, plasmids associated with antimicrobial resistance and virulence will likely be detected using hybrid assembly alone because these plasmids are usually larger in size.

## Methods

### Sample collection and isolation of *E. Coli* from diseased and healthy broilers

There is an existing program in Saskatchewan where producers bring dead broilers to have them analyzed by the Poultry Extension Service (PEX) at the University of Saskatchewan. Four- to six-week-old broiler chickens that had died on farms within a 4 h driving distance from Saskatoon were submitted to the PEX and necropsy was performed. Organ specimens were submitted for microbiological isolation and identification by MALDI-TOF mass spectrometry at Prairie Diagnostic Services (www.pdsinc.ca); a portion of the organs were stored at 4ºC until analysis was complete. If specimens were *E. coli* positive, PEX requested the submission of 3–4 healthy broilers from the same producer farms.

*E. coli* strains were isolated in the White lab from internal organs of diseased birds (liver, heart, spleen) that had been stored at 4ºC or from the cecal contents of healthy birds that were brought to the PEX. Equal sized pieces of each organ or cecal contents were placed in 2 mL SafeLock Eppendorf tubes with 1 mL of phosphate buffered saline (PBS) and a 5-mm steel bead (#69,989; Qiagen) and homogenized for 5 min at 30 Hz using a mixer mill (#MM400; Retsch). The homogenized solutions were serially diluted in PBS, plated on MacConkey Agar (BD Diagnostics) and grown overnight at 37ºC. Suspected *E. coli* colonies were confirmed using a positive indole and negative Simmons-citrate biochemical test [40]. Single colonies of confirmed *E. coli* were inoculated in 5ml of LB broth and incubated at 37ºC for 18 h with shaking; all isolates were stored in 50% glycerol at -80ºC for long-term preservation. We selected 12 ‘disease’ *E. coli* isolates and 7 ‘healthy’ isolates collected from 12 different boiler farms for plasmid analysis. These *E. coli* strains were part of larger cohort of 245 *E. coli* strains [22].

### Extraction of genomic DNA

*E. coli* isolates were streaked on Tryptic soy agar (TSA) from freezer stocks and grown at 37ºC for 18 h. A single colony was inoculated into 5 mL of LB broth and grown at 37ºC with shaking for 18 h. Genomic DNA was extracted from each *E. coli* isolate using the GenElute Bacterial Genomic DNA Extraction Kit (#NA2120; Millipore Sigma), as per manufacturer’s instructions. Purity of the DNA was assessed and quantified using a DeNovix spectrophotometer / fluorometer (#DS-11 FX; FroggaBio Inc.).

### Nanopore sequencing of the *E. Coli* isolates

#### Ligation procedure

All *E. coli* isolates were sequenced on a Nanopore MinION according to the protocol developed by Nick Lohman, Matt Loose, and Mick Watson for Porecamp Vancouver 2018 (http://porecamp.github.io). Prior to library preparation, DNA fragments > 500 bp were purified by addition of 0.4x (vol./vol.) NucleoMag NGS magnetic beads (#MN-744970.50; Macherey-Nagel). DNA was stored at -20ºC for up to one week before sequencing and stored at -80ºC for long term storage. Up to 200 fmol of purified and size-selected DNA (in a maximum of 24 µL) was added to an Eppendorf DNA Lobind microcentrifuge tube (#13-698-791; Fisher Scientific). Deionized water was added to a total volume of 30 µL. Nicks and gaps in the DNA were repaired using FFPE DNA Repair Mix (#M6630; NEB) and A-tails were added using the Ultra II End Repair/dA-Tailing Module (#E7645; NEB). Nanopore barcodes (#EXP-NBD104; ONT) were ligated to DNA fragments using the Blunt/TA Master Mix (#M0367; NEB). Multiplexed samples were pooled into a single Eppendorf tube and the pooled samples were adjusted to 1 M NaCl. DNA repair enzymes and excess DNA barcodes were removed using a 0.2X magnetic bead clean-up, which binds larger fragments of DNA. Sequencing adapters were ligated (#SQK-LSK110; ONT) to the ends of DNA fragments using the NEBnext quick ligation module (#E6056; NEB) before a final bead clean-up (1:1 ratio of beads to sample) was performed to generate the final purified DNA library. Beads were washed by incubating two times in long chain fragment buffer (LFB) and resuspended by flicking. DNA was eluted in 1mM Tris buffer, pH 8.0. Sequencing was performed using R9.4.1 MinION flow cells on the MinION Mk1C instrument. Sequencing runs were terminated after approximately 24 h.

#### Rapid barcoding procedure

Libraries were also prepared according to the rapid barcoding protocol (#SQK-RBK004; ONT). For each *E. coli* isolate, 500ng of purified DNA was transferred into a 0.2mL thin-walled PCR tube with the volume adjusted to 7.5uL using nuclease-free water. To each tube, 2.5uL of the fragmentation mix (#RB01-12) was added and incubated at 30ºC for 1 min; the transposase present cleaves the template DNA molecules and attaches the barcoded tags to the cleaved ends. The transposase was inactivated by incubating at 80ºC for 1 min. Multiplexed samples were pooled into a single Eppendorf tube and the pooled samples were adjusted to 1 M NaCl. Excess enzymes and DNA barcodes were removed using a 0.2X magnetic bead clean-up. 10uL of the barcoded DNA and 1uL of Rapid Sequencing Adapters (RAP) were added together in a new Lobind tube and incubated at room temperature for 5 min. Samples were sequenced using R9.4.1 MinION flow cells on the MinION Mk1C instrument. Sequencing runs were terminated after approximately 24 h.

### Illumina sequencing of the *E. coli* Isolates

Purified genomic DNA was used for Illumina library preparation, following steps according to the NEBNext Ultra II DNA Library Prep Kit protocol (#E7103; NEB). Approximately 100ng of genomic DNA was sheared by incubating the DNA with the NEBNext Ultra II FS Enzyme Mix (#E7805L; NEB) in a thermocycler for 12 min at 37 °C, followed by 30 min at 65 °C. NEBNext Adaptors were ligated to the fragmented DNA using NEBNext Ultra II Ligation Mix (#E7595L; NEB) by incubating at 20ºC for 15 min. To purify the DNA and remove excess enzymes, 25 µL aliquots of NGS magnetic beads were added to the samples. Finally, DNA was enriched via PCR using NEBNext Multiplex Oligos for Illumina (NEB, #E6609) using the following thermocycler conditions: denature for 30 s at 98 °C, 4 cycles of denaturing at 98 °C for 10 s followed by annealing and extension at 65 °C for 75 s, and a final extension at 65 °C for five minutes. Sequencing was performed by Novogene Corporation Inc. (Sacramento, CA) using an Illumina HiSeq 400 or NovaSeq, with 150 bp paired-end sequencing (300 cycles).

### Preparation of samples from plasmid extraction kits for DNA sequencing

*Escherichia coli* isolates 4957-3S1 and 4957-C3 were streaked on LB agar from freezer stocks and a single colony was incubated overnight at 37ºC in LB broth with shaking for 16 h. Plasmid DNA was isolated according to the manufacturer’s instructions from six commercial plasmid extraction kits: (A) GenElute plasmid Miniprep kit (#PLN70; Millipore Sigma), (B) NucleoSpin mini kit (#740588.50; Machery-Nagel), (C) Presto mini plasmid kit (#PD100; Geneaid), (D) Monarch plasmid miniprep kit ((#T1010S; New England Biolabs), (E) Plasmid midi kit (#12,143; Qiagen) and (F) GeneJet plasmid miniprep kit (#K0502; Fisher Scientific). The DNA samples generated from the plasmid extraction kits were fragmented by Cup and Horn sonication with a high-intensity ultrasonic processor (Vibra-Cell, Danbury, CT) for one 30-second pulse, followed by 2 min on ice. DNA samples were assessed for quality, purity, and integrity using a DeNovix spectrophotometer /fluorometer and an Agilent 2100 Bioanalyzer with a High Sensitivity DNA chip (#5067–4626; Agilent Technologies). DNA libraries generated with the Ligation sequencing kit or Rapid Barcoding kit were sequenced using R9.4.1 MinION flow cells on the MinION Mk1C instrument; runs were terminated after 24 h. For the other 17 *E. coli* isolates, plasmid DNA was extracted using kits D and F (above), sonication was performed and the ONT Ligation Sequencing Kit was used to prepare libraries for sequencing.

### DNA sequence assemblies

For each *E. coli* isolate, we generated hybrid assemblies, long read assemblies and plasmid assemblies. For Illumina, raw sequence reads were obtained directly from Novogene and were trimmed to remove the adapter sequences, using fastp (v0.20.1) [41]. The quality of Illumina reads was analyzed by FastQC (v0.11.9) [42] and contamination was determined using Confindr (v0.7.4) [43]. For Nanopore, raw sequence reads were obtained in-house with base calling performed using MinKNOW v2.0 [44] and Guppy v2.1.1 [45]. The quality of reads was analyzed by NanoStat (v1.5.0) [46]. Adapter sequences were trimmed from the Nanopore reads using Porechop (v0.2.4) and filtered with a minimum length of 1000 bp using filtlong (v0.2.1) or were left unfiltered.

Long read assemblies were generated from the Nanopore sequences using the Flye program (v2.9) [47]. The standard protocol was to polish these assemblies with long reads by Medaka (v1.7.2). For *E. coli* isolates where Unicycler failed to generate a closed hybrid assembly, Flye software was used to generate long read assemblies, which were polished up to 30 times with short reads using Pilon (v1.4) [48] and with long reads using Medaka. These long read first hybrid assemblies were compared to hybrid assemblies generated by Unicycler in a variety of tests.

For standard hybrid assemblies, the Unicycler (v0.5.0) [18] program compiled the Illumina and Nanopore reads to generate whole genome sequence assemblies using the long read assemblies as trusted contigs. Hybrid assemblies were polished by mapping the reads to the assembly and taking the consensus of the reads using Pilon (v1.24) (with MUMmer (v4.0.0) and SAMtools (v1.13)). Assemblies that still possessed SNPs (differences between the assembly and reads) were further polished using Snippy (v4.6.0) (with VCFtools ( v0.1.16) and Tabix (v0.2.6)), followed by another 15 rounds of polishing using Pilon. Hybrid and long read assemblies were confirmed to be from the same isolates using MLST (v2.22.1) and EZClermont (v0.7.0) [49]. The quality of assemblies was determined using Quast (v5.0.2) [22].

For plasmid DNA assemblies, the adapter sequences were trimmed from the Nanopore raw reads using Porechop and the trimmed reads were assembled using Canu (v2.2) [50]. The plasmid Nanopore reads were not filtered because this would exclude small plasmids, which could be represented by shorter reads.

### Plasmid identification using MOB-suite

The hybrid, long read and plasmid DNA assemblies were analyzed using MOB-suite [20]. The MOB-suite pipeline (v3.1.0) scans input assemblies for contigs containing plasmid-related genes (e.g., relaxases and replicases) and repetitive regions, thereby identifying putative plasmid scaffolds. These scaffolds are compared against a database of mobility clusters (MOB-clusters) comprising pre-clustered reference plasmids. The putative plasmids were assigned to MOB-clusters by identifying the minimum Mash distance to a reference plasmid in the database. The output consists of the contig sequences belonging to the MOB-clusters and an annotation of their host-range predictions, mobility predictions, and assignment to a replicase (rep) gene cluster. The results from MOB-suite from all the assemblies were amalgamated, processed, and analyzed in Python using packages including pandas (v1.0.3) to generate a presence/absence table and summary tables. All strip plots, bar plots and cluster maps were created in python using Pandas and Seaborn (v0.11.2) then retouched in Prism (v8.4.2) or Adobe Elements 2020. To determine the number of ORFs in each plasmid cluster detected in each isolate, we used ORFfinder v1.8 [51].

### Statistical analysis

Using the statannotations (v0.4.3) Python package, the relationships between different assembly types were analyzed using two-way ANOVAs with type three sum of squares and using a posthoc t-test with bonferroni correction. Comparisons of the assemblies were represented as strip plots and differences between assembly types were determined using independent t-tests. The scripts that were used to do the statistical analysis are found on our research group’s GitHub (https://github.com/Aaron-White-Lab/2022_Plasmids_Detection_Methods/).

### Supplementary Information

**Additional file 1: Table S2.** Detailed MOBsuite data from 19 poultry-associated E. coli strains from Saskatchewan.

**Additional file 2: Figure S1.** Quality control parameters determined for different types of hybrid genome assembly. Strip plots show parameter values for N50 (A), number of contigs (B), largest contig (C), total length (D) and GC content (E), for hybrid WGS assemblies from 19 *E. coli *isolates, as determined by Quast. Bars represent the mean parameter values corresponding to each type of hybrid assembly: 1) HLF, Illumina + Nanopore Ligation + filtered; 2) HLUF, Illumina + Nanopore Ligation + unfiltered; 3) HRF, Illumina + Nanopore Rapid + filtered; and 4) HRUF, Illumina + Nanopore Rapid + unfiltered. There were no significant differences in quality parameters between each assembly type as determined by t-test (*P *> 0.05). **Figure S2.** Quality control parameters determined for different types of long-read genome assembly.Box and whisker plots show parameter values for N50 (A), number of contigs (B), largest contig (C), total length (D) and GC content (E), for long read WGS assemblies from 19 *E. coli *isolates, as determined by Quast. The line in each box represents the mean parameter value corresponding to each type of long-read (L) assembly: 1) LLF, Nanopore Ligation+ filtered; 2) LLUF, Nanopore Ligation + unfiltered; 3) LRF, Nanopore Rapid + filtered; and 4) LRUF, Nanopore Rapid + unfiltered. Statistical significance in any pairwise comparison was determined using ttests (*P* < 0.05, *; *P* < 0.01,**). All other pairwise comparisons were not significant (*P* > 0.05). **Figure S3. **The effect of polishing long read sequence assemblies on plasmid detection by MOB-suite.The total numbers of detected plasmids are shown for long-read DNA sequence assemblies (Nanopore Ligation or Rapid kits, filtered or unfiltered) prepared from 19 *E. coli *isolates. The assemblies were either unpolished, polished with raw Nanopore reads, or the long-read assemblies were polished with Illumina reads to generate a hybrid assembly, prior to analysis by MOB-suite software. The values were compared between groups using t-tests and determined not to be significantly different (*P *> 0.05, ns). **Table S1.** DNA sequencing parameters for plasmid extraction kit samples prepared from two avian-associated *E. coli *isolates.

## Data Availability

Illumina and Nanopore raw sequence reads and corresponding hybrid whole genome assemblies are available as part of the NCBI Bioproject: PRJNA912639. Long-read whole genome assemblies, as well as raw reads and assemblies pertaining to the plasmid extractions are available upon request. The Tables and scripts used for analysis have been deposited at: https://github.com/Aaron-White-Lab/2022_Plasmids_Detection_Methods.

## Abbreviations

APEC: Avian pathogenic E. coli
AMR: Antimicrobial resistance
ONT: Oxford Nanopore Technology
LSK: Ligation sequencing kit
RB: Rapid barcoding kit
WGS: Whole genome sequencing
PEX: Poultry Extension Service
MALDI-TOF: Matrix-assisted laser desorption/ionization-time of flight

## References

[1] Newman DM, Barbieri NL, de Oliveira AL, Willis D, Nolan LK, Logue CM (2021). Characterizing avian pathogenic Escherichia coli (APEC) from colibacillosis cases, 2018. PeerJ.

[2] Guabiraba R, Schouler C (2015). Avian colibacillosis: still many black holes. FEMS Microbiol Lett.

[3] Mageiros L, Méric G, Bayliss SC, Pensar J, Pascoe B, Mourkas E, Calland JK, Yahara K, Murray S, Wilkinson TS, Williams LK (2021). Genome evolution and the emergence of pathogenicity in avian Escherichia coli. Nat Commun.

[4] Schouler C, Schaeffer B, Brée A, Mora A, Dahbi G, Biet F, Oswald E, Mainil J, Blanco J, Moulin-Schouleur M (2012). Diagnostic strategy for identifying avian pathogenic Escherichia coli based on four patterns of virulence genes. J Clin Microbiol.

[5] Delgado-Blas JF, Ovejero CM, David S, Montero N, Calero-Caceres W, Garcillan-Barcia MP, de la Cruz F, Muniesa M, Aanensen DM, Gonzalez-Zorn B (2021). Population genomics and antimicrobial resistance dynamics of Escherichia coli in wastewater and river environments. Commun Biology.

[6] Johnson TJ, Siek KE, Johnson SJ, Nolan LK (2006). DNA sequence of a ColV plasmid and prevalence of selected plasmid-encoded virulence genes among avian Escherichia coli strains. J Bacteriol.

[7] Mellata M, Ameiss K, Mo H, Curtiss R (2010). Characterization of the contribution to virulence of three large plasmids of avian pathogenic Escherichia coli χ7122 (O78: K80: H9). Infect Immun.

[8] Skyberg JA, Siek KE, Doetkott C, Nolan LK (2007). Biofilm formation by avian Escherichia coli in relation to media, source and phylogeny. J Appl Microbiol.

[9] Ogura Y, Ooka T, Iguchi A, Toh H, Asadulghani M, Oshima K, Kodama T, Abe H, Nakayama K, Kurokawa K, Tobe T (2009). Comparative genomics reveal the mechanism of the parallel evolution of O157 and non-O157 enterohemorrhagic Escherichia coli. Proc Natl Acad Sci.

[10] Phan MD, Kidgell C, Nair S, Holt KE, Turner AK, Hinds J, Butcher P, Cooke FJ, Thomson NR, Titball R, Bhutta ZA (2009). Variation in Salmonella enterica serovar typhi IncHI1 plasmids during the global spread of resistant typhoid fever. Antimicrob Agents Chemother.

[11] Thomas CM, Summers D (2008). Bacterial plasmids. ELS.

[12] Gillings MR, Stokes HW (2012). Are humans increasing bacterial evolvability?. Trends Ecol Evol.

[13] Heuer H, Smalla K (2012). Plasmids foster diversification and adaptation of bacterial populations in soil. FEMS Microbiol Rev.

[14] Thakur S, Gray GC (2019). The mandate for a global “one health” approach to antimicrobial resistance surveillance. Am J Trop Med Hyg.

[15] De Maio N, Shaw LP, Hubbard A, George S, Sanderson ND, Swann J, Wick R, AbuOun M, Stubberfield E, Hoosdally SJ, Crook DW. Comparison of long-read sequencing technologies in the hybrid assembly of complex bacterial genomes. Microb Genomics. 2019;5(9). 10.1099/mgen.0.000294. 10.1099/mgen.0.000294 PMC6807382 31483244

[16] Sereika M, Kirkegaard RH, Karst SM, Michaelsen TY, Sørensen EA, Wollenberg RD, Albertsen M (2021). Oxford Nanopore R10. 4 long-read sequencing enables near-perfect bacterial genomes from pure cultures and metagenomes without short-read or reference polishing. Microbiology.

[17] Berbers B, Saltykova A, Garcia-Graells C, Philipp P, Arella F, Marchal K, Winand R, Vanneste K, Roosens NH, De Keersmaecker SC (2020). Combining short and long read sequencing to characterize antimicrobial resistance genes on plasmids applied to an unauthorized genetically modified Bacillus. Sci Rep.

[18] McArthur AG, Tsang KK (2017). Antimicrobial resistance surveillance in the genomic age. Ann N Y Acad Sci.

[19] Wick RR, Judd LM, Gorrie CL, Holt KE (2017). Unicycler: resolving bacterial genome assemblies from short and long sequencing reads. PLoS Comput Biol.

[20] Wick RR, Holt KE, Zimin A, Salzberg SL, Hopkins J, Vaser R. Benchmarking of long-read assemblers for prokaryote whole genome sequencing. F1000Res. 8:2138. 10.12688/f1000research.21782.1. 10.12688/f1000research.21782.1 PMC6966772 31984131

[21] Robertson J, Nash JH. MOB-suite: software tools for clustering, reconstruction and typing of plasmids from draft assemblies. Microbial genomics. 2018;4(8). .10.1099/mgen.0.000206 10.1099/mgen.0.000206 PMC6159552 30052170

[22] Gurevich A, Saveliev V, Vyahhi N, Tesler G (2013). QUAST: quality assessment tool for genome assemblies. Bioinformatics.

[23] Sanderson H, Nnajide CR, McCarthy MC, Singh R, Rubin JE, Dillon JA, White AP (2023). Hybrid genome assemblies of 245 avian and broiler barn environment-associated Escherichia coli strains isolated from Saskatchewan Broiler Farms. Microbiol Resour Announc.

[24] Khezri A, Avershina E, Ahmad R (2021). Hybrid assembly provides improved resolution of plasmids, antimicrobial resistance genes, and virulence factors in Escherichia coli and Klebsiella pneumoniae clinical isolates. Microorganisms.

[25] Johnson TJ (2021). Role of plasmids in the ecology and evolution of “High-Risk” extraintestinal pathogenic Escherichia coli clones. EcoSal Plus.

[26] Doetkott DM, Nolan LK, Giddings CW, Berryhill DL (1996). Large plasmids of avian Escherichia coli isolates. Avian Dis.

[27] Olsen RH, Christensen H, Bisgaard M (2012). Comparative genomics of multiple plasmids from APEC associated with clonal outbreaks demonstrates major similarities and identifies several potential vaccine-targets. Vet Microbiol.

[28] Mellata M, Maddux JT, Nam T, Thomson N, Hauser H, Stevens MP, Mukhopadhyay S, Sarker S, Crabbe A, Nickerson CA, Santander J, Curtiss R (2012). New insights into the bacterial fitness-associated mechanisms revealed by the characterization of large plasmids of an avian pathogenic E. Coli. PLoS One.

[29] George S, Pankhurst L, Hubbard A, Votintseva A, Stoesser N, Sheppard AE, Mathers A, Norris R, Navickaite I, Eaton C, Iqbal Z, Crook DW, Phan HTT (2017). Resolving plasmid structures in *Enterobacteriaceae* using the MinION nanopore sequencer: assessment of MinION and MinION/Illumina hybrid data assembly approaches. Microb Genom.

[30] Ferreira GN, Monteiro GA, Prazeres DM, Cabral JM (2000). Downstream processing of plasmid DNA for gene therapy and DNA vaccine applications. Trends Biotechnol.

[31] Shareck J, Choi Y, Lee B, Miguez CB (2004). Cloning vectors based on cryptic plasmids isolated from lactic acid bacteria: their characteristics and potential applications in biotechnology. Crit Rev Biotechnol.

[32] Prazeres DM, Monteiro GA. Plasmid biopharmaceuticals. Plasmids: Biology and impact in biotechnology and discovery. 2015:669-88. 10.1128/9781555818982.ch34.

[33] Wick RR, Judd LM, Wyres KL, Holt KE. Recovery of small plasmid sequences via Oxford Nanopore sequencing. Microb Genomics. 2021;7(8). 10.1099/mgen.0.000631. 10.1099/mgen.0.000631 PMC8549360 34431763

[34] Johnson TJ, Nolan LK (2009). Pathogenomics of the virulence plasmids of Escherichia coli. Microbiol Mol Biol Rev.

[35] Papoušková A, Čížek A (2020). A complex approach to a complex problem: the use of whole-genome sequencing in monitoring avian-pathogenic Escherichia coli–a review. Acta Vet Brno.

[36] Tyson GH, McDermott PF, Li C, Chen Y, Tadesse DA, Mukherjee S, Bodeis-Jones S, Kabera C, Gaines SA, Loneragan GH, Edrington TS (2015). WGS accurately predicts antimicrobial resistance in Escherichia coli. J Antimicrob Chemother.

[37] Sanderson H, Gray KL, Manuele A, Maguire F, Khan A, Liu C, Navanekere Rudrappa C, Nash JH, Robertson J, Bessonov K, Oloni M (2022). Exploring the mobilome and resistome of Enterococcus faecium in a one health context across two continents. Microb Genomics.

[38] Yin D, Cheng B, Yang K, Xue M, Lin Y, Li Z, Song X, Shao Y, Tu J, Li P, Qi K (2021). Complete genetic analysis of plasmids carrying mcr-1 and other resistance genes in avian pathogenic Escherichia coli isolates from diseased chickens in Anhui province in China. Msphere.

[39] Li R, Xie M, Lv J, Wai-Chi Chan E, Chen S (2017). Complete genetic analysis of plasmids carrying mcr-1 and other resistance genes in an Escherichia coli isolate of animal origin. J Antimicrob Chemother.

[40] Vaughn RH, Osborne JT, Wedding GT, Tabachnick J, Beisel CG, Braxton T (1950). The utilization of citrate by Escherichia coli. J Bacteriol.

[41] Chen S, Zhou Y, Chen Y, Gu J (2018). Fastp: an ultra-fast all-in-one FASTQ preprocessor. Bioinformatics.

[42] Andrews S, Krueger F, Segonds-Pichon A, Biggins L, Krueger C, Wingett S. FastQC. A quality control tool for high throughput sequence data. Babraham Institute; 2010;370.

[43] Low AJ, Koziol AG, Manninger PA, Blais B, Carrillo CD (2019). ConFindr: rapid detection of intraspecies and cross-species contamination in bacterial whole-genome sequence data. PeerJ.

[44] Oxford Nanopore Technologies. 2021. MinKNOW. https://github.com/nanoporetech/minknow_api.

[45] Oxford Nanopore Technologies. 2020. https://github.com/nanoporetech/pyguppyclient.

[46] De Coster W, D’hert S, Schultz DT, Cruts M, Van Broeckhoven C (2018). NanoPack: visualizing and processing long-read sequencing data. Bioinformatics.

[47] Kolmogorov M, Yuan J, Lin Y, Pevzner PA (2019). Assembly of long, error-prone reads using repeat graphs. Nat Biotechnol.

[48] Walker BJ, Abeel T, Shea T, Priest M, Abouelliel A, Sakthikumar S, Cuomo CA, Zeng Q, Wortman J, Young SK, Earl AM (2014). Pilon: an integrated tool for comprehensive microbial variant detection and genome assembly improvement. PLoS One.

[49] Waters NR, Abram F, Brennan F, Holmes A, Pritchard L. Easy phylotyping of Escherichia coli via the EzClermont web app and command-line tool. Access Microbiol. 2020;2(9). 10.1099/acmi.0.000143. 10.1099/acmi.0.000143 PMC7656184 33195978

[50] Koren S, Walenz BP, Berlin K, Miller JR, Bergman NH, Phillippy AM (2017). Canu: scalable and accurate long-read assembly via adaptive k-mer weighting and repeat separation. Genome Res.

[51] Rombel IT, Sykes KF, Rayner S, Johnston SA (2002). ORF-FINDER: a vector for high-throughput gene identification. Gene.

