# Supplementary material for: Identification of plasmids in avian-associated Escherichia coli using nanopore and illumina sequencing
Source: BMC Genomics. 2023 Nov 21;24:698. doi: 10.1186/s12864-023-09784-6 (PMC10664647; doi:10.1186/s12864-023-09784-6)
Supplement: Supplementary file 2 — Additional file 2: Figure S1. Quality control parameters determined for different types of hybrid genome assembly. Strip plots show parameter values for N50 (A), number of contigs (B), largest contig (C), total length (D) and GC content (E), for hybrid WGS assemblies from 19 E. coli isolates, as determined by Quast. Bars represent the mean parameter values corresponding to each type of hybrid assembly: 1) HLF, Illumina + Nanopore Ligation + filtered; 2) HLUF, Illumina + Nanopore Ligation + unfiltered; 3) HRF, Illumina + Nanopore Rapid + filtered; and 4) HRUF, Illumina + Nanopore Rapid + unfiltered. There were no significant differences in quality parameters between each assembly type as determined by t-test (P > 0.05). Figure S2. Quality control parameters determined for different types of long-read genome assembly.Box and whisker plots show parameter values for N50 (A), number of contigs (B), largest contig (C), total length (D) and GC content (E), for long read WGS assemblies from 19 E. coli isolates, as determined by Quast. The line in each box represents the mean parameter value corresponding to each type of long-read (L) assembly: 1) LLF, Nanopore Ligation+ filtered; 2) LLUF, Nanopore Ligation + unfiltered; 3) LRF, Nanopore Rapid + filtered; and 4) LRUF, Nanopore Rapid + unfiltered. Statistical significance in any pairwise comparison was determined using ttests (P < 0.05, *; P < 0.01,**). All other pairwise comparisons were not significant (P > 0.05). Figure S3. The effect of polishing long read sequence assemblies on plasmid detection by MOB-suite.The total numbers of detected plasmids are shown for long-read DNA sequence assemblies (Nanopore Ligation or Rapid kits, filtered or unfiltered) prepared from 19 E. coli isolates. The assemblies were either unpolished, polished with raw Nanopore reads, or the long-read assemblies were polished with Illumina reads to generate a hybrid assembly, prior to analysis by MOB-suite software. The values were compared between [file 12864_2023_9784_MOESM2_ESM.pdf]

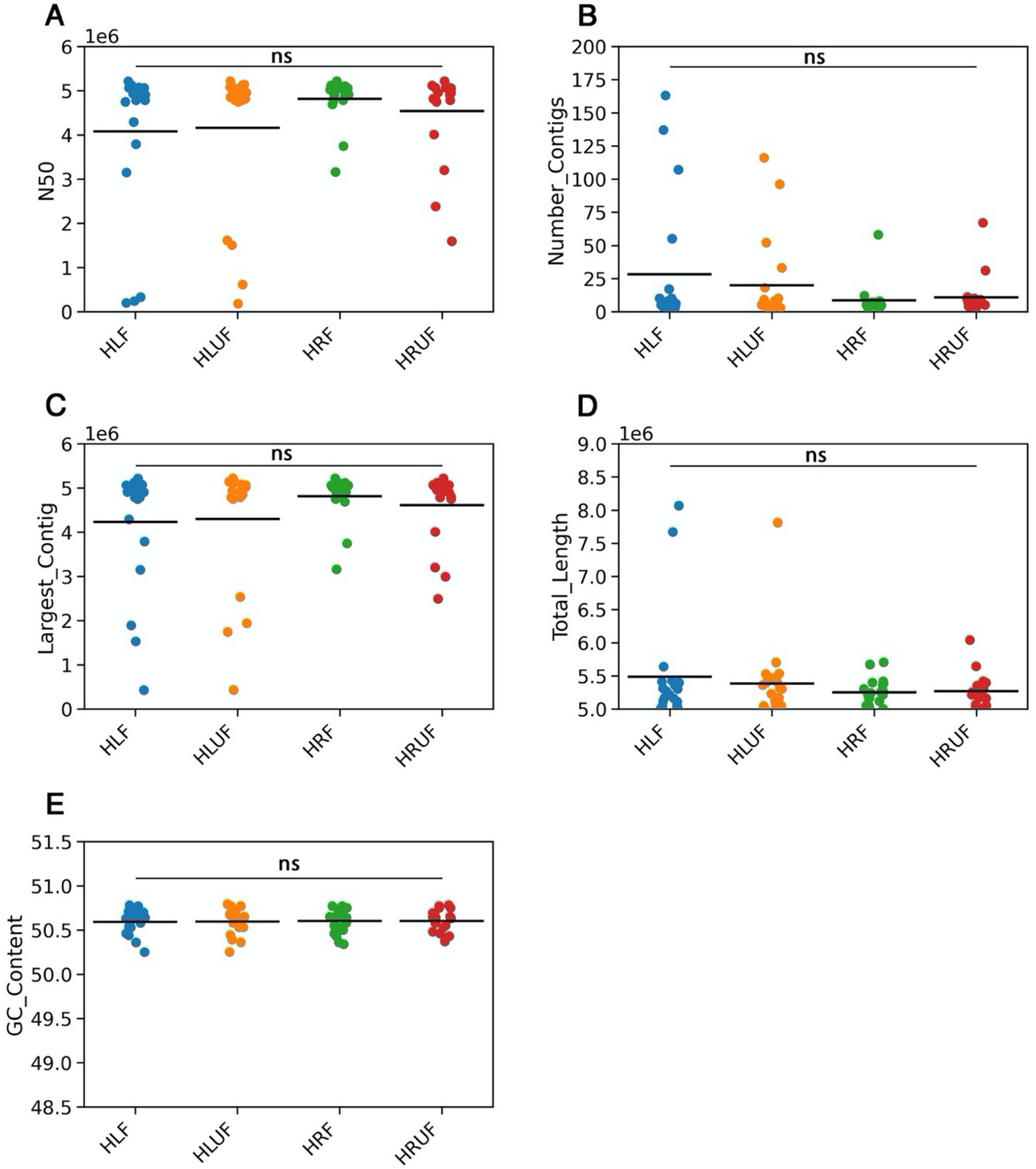


**Figure S1. Quality control parameters determined for different types of hybrid genome assembly.**

Strip plots show parameter values for N50 (A), number of contigs (B), largest contig (C), total length (D) and GC content (E), for hybrid WGS assemblies from 19 *E. coli* isolates, as determined by Quast. Bars represent the mean parameter values corresponding to each type of hybrid assembly: 1) HLF, Illumina + Nanopore Ligation + filtered; 2) HLUF, Illumina + Nanopore Ligation + unfiltered; 3) HRF, Illumina + Nanopore Rapid + filtered; and 4) HRUF, Illumina + Nanopore Rapid + unfiltered. There were no significant differences in quality parameters between each assembly type as determined by t-test ($P > 0.05$).

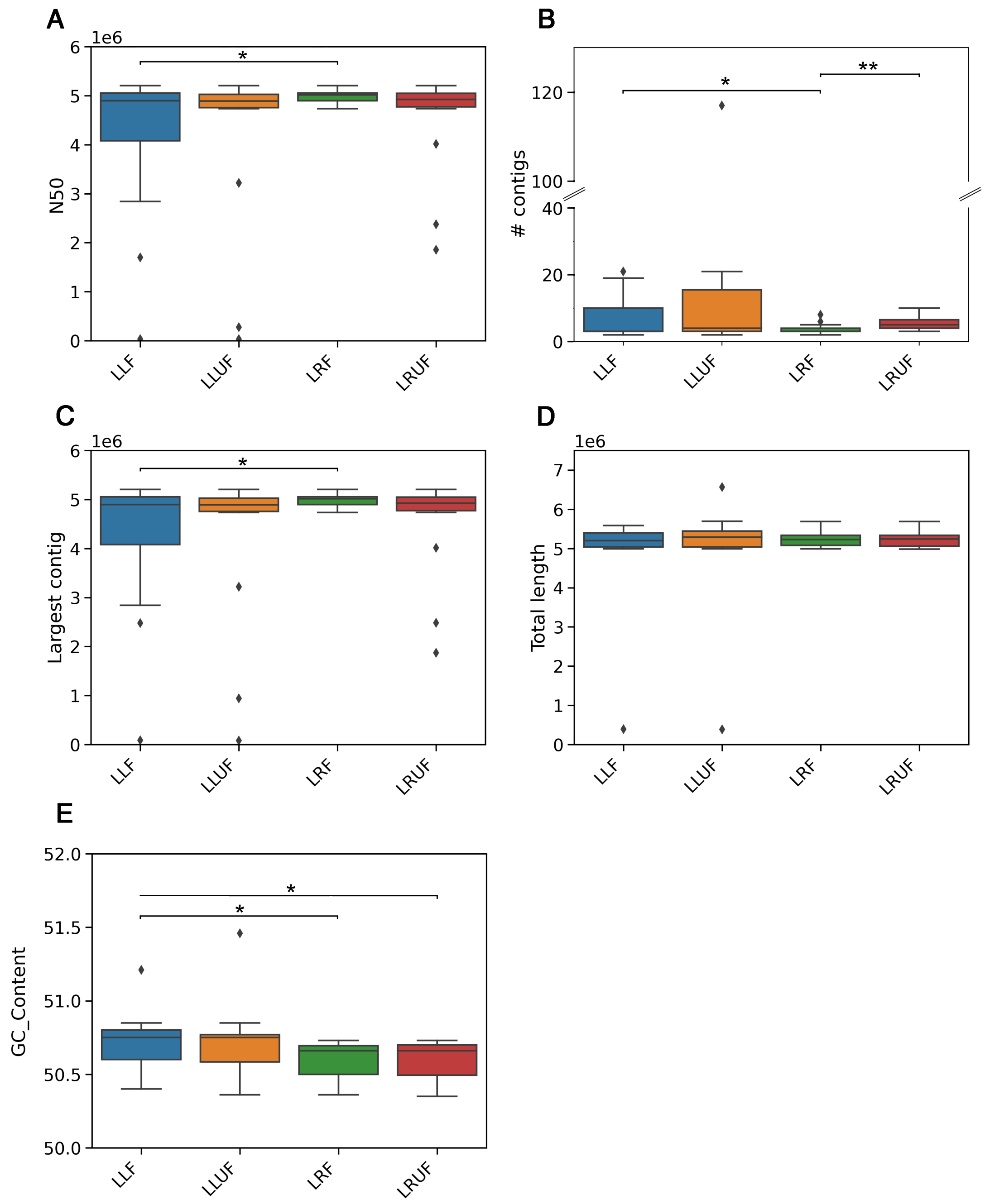


**Figure S2. Quality control parameters determined for different types of long-read genome assembly.**

Box and whisker plots show parameter values for N50 (A), number of contigs (B), largest contig (C), total length (D) and GC content (E), for long read WGS assemblies from 19 *E. coli* isolates, as determined by Quast. The line in each box represents the mean parameter value corresponding to each type of long-read (L) assembly: 1) LLF, Nanopore Ligation + filtered; 2) LLUF, Nanopore Ligation + unfiltered; 3) LRF, Nanopore Rapid + filtered; and 4) LRUF, Nanopore Rapid + unfiltered. Statistical significance in any pairwise comparison was determined using t-tests ($P < 0.05$, *; $P < 0.01$, **). All other pairwise comparisons were not significant ($P > 0.05$).

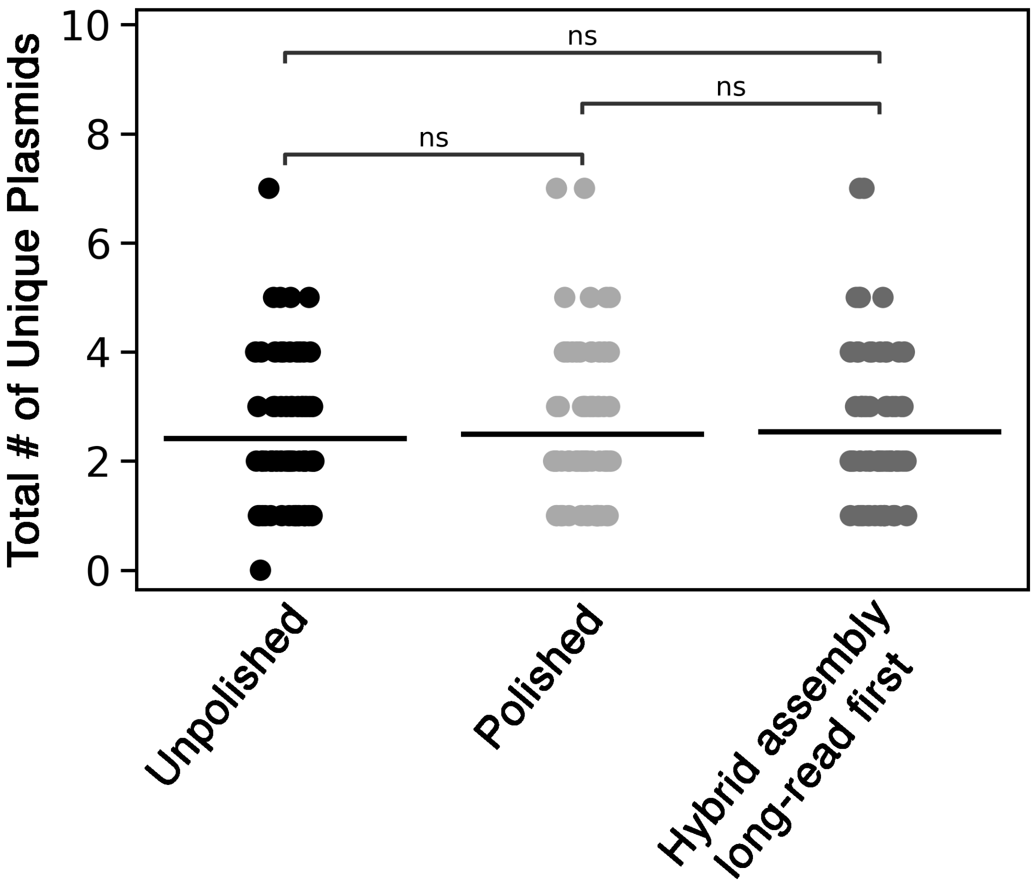


**Figure S3. The effect of polishing long read sequence assemblies on plasmid detection by MOB-suite.**

The total numbers of detected plasmids are shown for long-read DNA sequence assemblies (Nanopore Ligation or Rapid kits, filtered or unfiltered) prepared from 19 *E. coli* isolates. The assemblies were either unpolished, polished with raw Nanopore reads, or the long-read assemblies were polished with Illumina reads to generate a hybrid assembly, prior to analysis by MOB-suite software. The values were compared between groups using t-tests and determined not to be significantly different ($P > 0.05$, ns).

**Table S1. DNA sequencing parameters for plasmid extraction kit samples prepared from two avian-associated *E. coli* isolates.**

| | | Ligation Sequencing Kit | | | Rapid Barcoding Kit | | |
|---|---|---|---|---|---|---|---|
| *E. coli* Isolate | Plasmid Extraction Kit[a] | # Reads[b] | Ave. Length[b] (bp) | Quality[b] | # Reads[b] | Ave. Length[b] (bp) | Quality[b] |
| 4957-3S1 | A | 116,000 | 934 | 10.8 | 138,188 | 736 | 10.2 |
| 4957-3S1 | B | 32,000 | 1388 | 10.8 | 56,622 | 1173 | 10.2 |
| 4957-3S1 | C | 100,000 | 2172 | 10.7 | 49,787 | 1193 | 10.3 |
| 4957-3S1 | D | 20,000 | 3510 | 10.6 | 189,877 | 1660 | 10.3 |
| 4957-3S1 | E | 16,000 | 3832 | 9.5 | 184,000 | 3815 | 10.4 |
| 4957-3S1 | F | 80,000 | 4388 | 10.8 | 176,589 | 1250 | 10.3 |
| 4957-C3 | A | 400,000 | 1072 | 11.2 | 153,344 | 785 | 10.4 |
| 4957-C3 | B | 192,000 | 1104 | 11.1 | 13,108 | 1376 | 10.4 |
| 4957-C3 | C | 408,000 | 1620 | 11.2 | 165,547 | 1599 | 10.6 |
| 4957-C3 | D | 20,000 | 4726 | 10.8 | 268,113 | 2146 | 10.7 |
| 4957-C3 | E | 24,000 | 4918 | 10.4 | 348,287 | 2763 | 10.7 |
| 4957-C3 | F | 140,000 | 3093 | 11.1 | 145,569 | 1195 | 10.4 |

[a] Commercial plasmid extraction kits were used to prepare samples from the two *E. coli* isolates: A – GenElute plasmid miniprep kit (#PLN70; Millipore Sigma); B – NucleoSpin plasmid mini kit (#740588.50; Machery-Nagel); C – Presto mini plasmid kit (#PD100; Geneaid); D – Monarch plasmid miniprep kit (#T1010S; New England Biolabs); E – Plasmid midi kit (#12143; Qiagen); and F – GeneJET plasmid miniprep kit (#K0502; ThermoFisher)

[b] Number of reads, average DNA fragment length and overall quality was determined by NanoStat (v1.5.0).
